# Quantifying Pathological Synergies in the Upper Extremity of Stroke Subjects With the Use of Inertial Measurement Units: A Pilot Study

**DOI:** 10.1109/JTEHM.2020.3042931

**Published:** 2020-12-07

**Authors:** Miguel M. C. Bhagubai, Gerjan Wolterink, Anne Schwarz, Jeremia P. O. Held, Bert-Jan F. Van Beijnum, Peter H. Veltink

**Affiliations:** 1 Biomedical Signals and Systems~(BSS) Research GroupUniversity of Twente 3230 7522LWEnschedeThe Netherlands; 2 Robotics and Mechatronics GroupUniversity of Twente 3230 7522NHEnschedeThe Netherlands; 3 Division of Vascular Neurology and NeurorehabilitationDepartment of NeurologyUniversity Hospital Zürich, University of Zürich 27243 8091ZürichSwitzerland

**Keywords:** FMA, IMU, kinematics, motor-synergy, stroke

## Abstract

Background: Stroke is one of the main causes of disability in the world, causing loss of motor function on mainly one side of the body. A proper assessment of motor function is required to help to direct and evaluate therapy. Assessment is currently performed by therapists using observer-based standardized clinical assessment protocols. Sensor-based technologies can be used to objectively quantify the presence and severity of motor impairments in stroke patients. Methods: In this work, a minimally obstructive distributed inertial sensing system, intended to measure kinematics of the upper extremity, was developed and tested in a pilot study, where 10 chronic stroke subjects performed the arm-related tasks from the Fugl-Meyer Assessment protocol with the affected and non-affected side. Results: The pilot study showed that the developed distributed measurement system was adequately sensitive to show significant differences in stroke subjects’ arm postures between the affected and non-affected side. The presence of pathological synergies can be analysed using the measured joint angles of the upper limb segments, that describe the movement patterns of the subject. Conclusion: Features measured by the system vary from the assessed FMA-UE sub-score showing its potential to provide more detailed clinical information.

## I. Introduction

Stroke is the third most common cause of disability in the world [1]. Approximately 1.1 million people in Europe [2] and eight hundred thousand people in the United States of America [3] suffer from stroke each year. Up to 50% of stroke survivors become chronically disabled [4], and experience impairments related to upper limb motor function [5], [6], such as muscle weakness or paresis, spasticity and decreased inter-joint coordination [7].

The normal muscle co-activation patterns exist in a stable spatiotemporal way across different muscles, and work in the sense of performing complex functional movements. The dependent pattern of muscle recruitment and activation is known as motor-synergy. In stroke, the damaged brain cells cause an interruption of the neural pathways. When the cortical cells reorganize, alternative descending pathways emerge [8]. The rearrangement of the descending motor neurons may result in an inevitable joint excitation or inhibition of different muscles. The abnormal co-activation muscle patterns are known as pathological synergies, and are associated with a reduced number of degrees of freedom of the motor control [9]. The pathological synergistic movement in stroke patients have been described in the past by Twitchell [10] and Brunnstrom [11]. In the process of motor function recovery, voluntary movement is characterized by two main muscle-coupling-patterns: the flexor and the extensor synergies. In the flexor synergy, an attempt of movement results in a coupled abduction and external rotation of the shoulder, flexion of the elbow, wrist and fingers, and forearm supination. Similarly, the extensor synergy is characterized by a coupled adduction and internal rotation of the shoulder, elbow extension, wrist and finger flexion, and forearm pronation [9]. These synergy patterns were objectively characterized by measuring the joint torques produced in the upper arm of patients by Dewald *et al.* [12]. Furthermore, these impairments create a learned bad use of the affected extremity, where compensation strategies are adopted by stroke survivors to increase success in completion of tasks [7], [13].

The importance of assessing motor function rises with the need of proper rehabilitation methods. Motor outcomes due to stroke, and its extent, differ between patients. A subject-specific rehabilitation protocol is needed, and frequent assessment provides better adaptation to the patient’s progress [14]. One of the most used assessment scales in the clinic to evaluate motor function is the Fugl-Meyer Assessment (FMA) [15]. The FMA was developed with the foundations of the motor recovery stages described by Twitchell and Brunnstrom [16]. Although it has been used as a gold standard method of upper extremity motor function assessment, it is an ordinal scale with insufficient sensitivity and suffers from a ceiling effect when evaluating patients with mild impairment [17]. Despite the high values for reliability and validity, the assessment procedure and scoring requirements have to be well defined in order to correctly assess the patient and avoid subjective variation in the score depending on the assessor.

Recently, kinematic measurements have been used to objectively quantify motor function in the upper extremity of stroke patients. Kinematic assessment can provide several metrics and features that allow an objective evaluation of both motor function and performance [18], [19]. A wide variety of kinematic measurement systems are used. Optical motion capture systems are considered the gold standard system to measure body kinematics [20]–[22]. However, the high cost and the need of a customized laboratory with fixed cameras are the main limitations. Robotic systems with motion capture capabilities such as exoskeletons and end-effectors in combination with arm weight support to control shoulder abduction loading, have also been used [23]–[27]. These robotic systems obstruct and have influence on the movement, reducing degrees of freedom in some cases. A widely used kinematic type of sensors are inertial measurement units (IMU). These sensors are low-cost and portable, and do not need specialized laboratories to perform the measurements. The majority of the studies that use wearable IMUs evaluate the relation between daily living tasks and clinical assessment scales, focusing on performance evaluation [18], [19], [28], [29]. Objective assessment of the actual FMA upper extremity subscale (FMA-UE) has been done before with accelerometers and gyroscopes [30], but the extracted features did not take in account the sensor’s orientation estimate and measurements of joint angles of the different limb segments. The purpose of the distributed sensing system is also to aid clinicians in their evaluation. An objective measurement of the arm joint angles is expected to be easily translatable to the clinic and understood by the therapist.

In this pilot study, a custom upper limb IMU system was developed and used to measure kinematics from, the sternum to the finger tips, of stroke patients while performing items from the FMA-UE. The goal of this study is to assess the capability of a new IMU system in evaluating motor function of the upper limb of stroke patients and comparing the pathological with non-pathological movement patterns. It is hypothesized that the kinematic features measured with the distributed IMU system can objectively distinguish the arm posture of the affected and the non-affected arms of stroke patients when the clinical evaluation tasks are performed. Based on knowledge of the pathological muscle coupling after stroke, it is expected that patients have an increased difficulty in reaching the desired target arm posture during the FMA-UE, when performed with the affected arm. The characteristics of the flexor and extensor synergies, such as stronger coupling between the shoulder, elbow and wrist, are also expected to be present on the affected arm’s movement profile. Another aim of this study is to evaluate if the system can distinguish more affected from less affected patients based on the FMA-UE score given by the therapist.

## II. Methodology

The methods adopted in this work align towards a detailed analysis of the upper limb’s movement of a patient. This is accomplished by using a measuring system composed of an increased number of sensors designed to provide a full reconstruction of the upper limb segments whilst being minimally obstructive and portable. The kinematic analysis is of relatively short lasting movements only using inertial sensors, not applying magnetometers due to the sensitivity to disturbances.

### A. Measurement System

**FIGURE 1.**
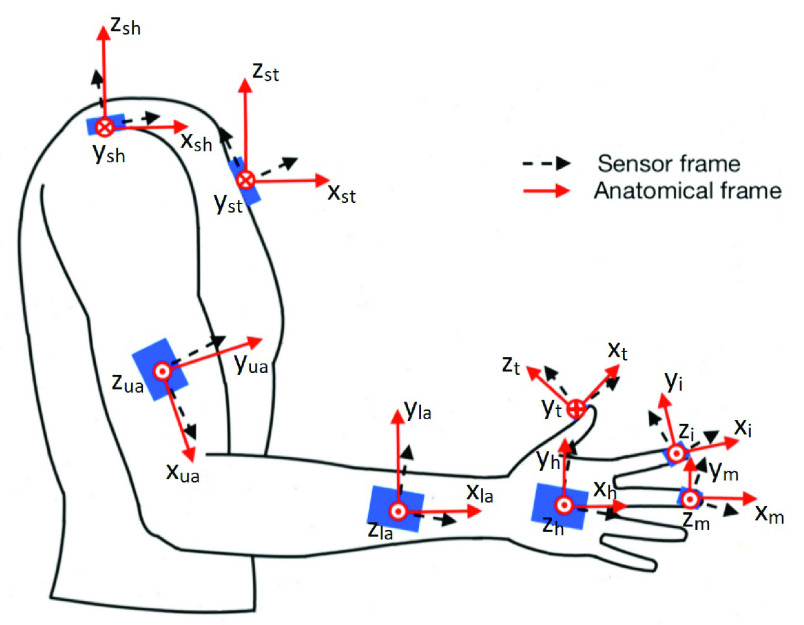
Sensor’s frames and correspondent anatomical frames of the eight different IMUs of the measurement system placed on the right arm.

The used distributed experimental sensing system is composed of eight IMUs (See Figure 1). It was based on the previous developed system by Kortier *et al.* [31]. The system consists of multiple small rigid printed circuit boards (PCB) that are interconnected by flexible cabling, where each rigid section contains a pair of triaxial gyroscopes and accelerometers (ST LSM330DLC). The system is divided into an arm and a hand substring. The arm string consists of four IMUs that are mounted on and measure the kinematics of the sternum, shoulder, upper arm and lower arm. The hand string has IMUs that are mounted on the dorsal side of the hand, thumb, index and middle fingers. A microcontroller (Atmel XMEGA) is responsible for data collection and for the USB interface to the computer. The data is collected at sampling frequency of 200 Hz for the gyroscopes and 100 Hz for the accelerometers.

The IMUs are covered by a 3D-printed Thermoplastic polyurethane (TPU) housing and were mounted to the respective body segment using 3D-printed flexible mounting straps. The shoulder and sternum IMU housings were fixed using medical tape. The sternum IMU was placed at the centre of the chest; the shoulder sensor was placed between the superior border of the scapula and the clavicle, near the acromioclavicular joint; the upper arm IMU was placed on the lateral side of the upper arm, near the elbow; the lower arm sensor was placed on the dorsal side of the forearm close to the wrist; the hand IMU was placed on the dorsal side of the hand, roughly in the centre; the finger sensors were placed in the distal inter phalanges of the thumb, index and middle finger.

### B. Kinematic Reconstruction

Since the system measures accelerations and angular velocities, several steps are needed to estimate the orientation of each sensor, and therefore, each body segment.

#### 1) Sensor-to-Segment Calibration

First, a sensor-to-segment calibration was conducted in order to find the orientation of the sensor with respect to the corresponding body segment. The sensor’s and the anatomical frames defined are shown in Figure 1. The coordinate systems of the sternum, arm and hand are arbitrarily defined in a way that the x-axis of the anatomical frame is in the longitudinal direction of the segment. The alignment of the sensors to the respective limb segments is based on the acquisition of data while the subject is in defined static postures or during dynamic movements. The principle is that measurements of the gravity vector with the accelerometer when the limb segment is in a defined posture represents an axis of the anatomical frame of the respective limb; likewise, measurements of the sensor’s angular velocity during a defined dynamic movement also represent an axis of the anatomical frame of one limb segment. This protocol was based on the work of Luinge *et al.* [32] and Ricci *et al.* [33]. The description of each posture/movement and the anatomical axis that are defined with each particular calibration movement can be found in Table 1. For each sensor, two different axes were measured with the sensors (depending on the segment, either by the accelerometer or the gyroscope), and the third axis was calculated using the cross-product of the previous two axes. Subsequently, one of the first two axes was redefined to ensure orthonormality in the coordinate system.

**TABLE 1:** Anatomical Axes of the Left and Right Arms Defined by Each Item of the Sensor-to-Segment Calibration Protocol

	Calibration Position/Movement	Anatomical Axis Left Arm	Anatomical Axis Right Arm
1	Static hand flat on a table	$\vec {z_{h}}, \vec {z_{i}}, \vec {z_{m}}$	$\vec {z_{h}}, \vec {z_{i}}, \vec {z_{m}}$
2	Static hand sideways, with elbow flexed 90°	$-\vec {y_{h}}, -\vec {y_{i}}, -\vec {y_{m}}$	$\vec {y_{h}}, \vec {y_{i}}, \vec {y_{m}}$
3	Static thumb flat on a table	$\vec {z_{t}}$	$\vec {z_{t}}$
4	Static thumb sideways, with hand pronated	$\vec {y_{t}}$	$-\vec {y_{t}}$
5	Static forearm and hand palm faced down	$\vec {z_{la}}$	$\vec {z_{la}}$
7	Wrist pronation, starting form supinated position	$\vec {x_{la}}$	$-\vec {z_{la}}$
8	Static shoulder adducted, with elbow flexed	$-\vec {x_{ua}}$	$-\vec {x_{ua}}$
8	Static shoulder abducted 90° with elbow flexed	$\vec {z_{ua}}$	$\vec {z_{ua}}$
9	Static straight neutral pose, arm along the body	$\vec {z_{sh}}, \vec {z_{st}}$	$\vec {z_{sh}}, \vec {z_{st}}$
10	Hip flexion (arm moving along the upper body)	$\vec {y_{sh}}, \vec {y_{st}}$	$-\vec {y_{sh}}, -\vec {y_{st}}$

A median filter was applied to the accelerometer data during the static positions or to the gyroscope data during the dynamic movements in order to get the anatomical frame relative to the sensor’s frame:

\begin{equation*} \vec {v_{seg}} = \frac {median(\vec {a_{s}})}{||median(\vec {a_{s}})||} \quad \text {or}~\vec {v_{seg}} = \frac {median(\vec {\omega _{s}})}{||median(\vec {\omega _{s}})||}\tag{1}\end{equation*}

where $\vec {v_{seg}}$ is the anatomical axis relative to the sensor $s$, and $\vec {a_{s}}$ and $\vec {\omega _{s}}$ are the accelerometer and gyroscope measurements in the respective calibration static position or dynamic movement respectively. The orientation of the segments’ coordinate frame relative to the sensor’s coordinate frame is given by a rotation matrix that contains three vectors that correspond to the anatomical axes of the segment, expressed in the sensor coordinate system:

\begin{equation*} ^{S}\textrm {R}_{Seg} = [\vec {x_{seg}}~\vec {y_{seg}}~\vec {z_{seg}}]\tag{2}\end{equation*}

#### 2) Global Frame Definition

In order to relate the orientations of the segments to each other, needed for the kinematic reconstruction of the upper extremity, a common global frame needs to be defined. For this definition, the last two movements (static neutral pose and hip flexion) of the sensor-to-segment calibration protocol were used [34]. The static neutral pose, with the arm stretched along the body and the fingers extended, is used to define the common vertical axis by measuring the gravity vector in all sensors. The hip flexion movement is performed with the arms stretched and accompanying the trunk movement. The measured angular velocity is used to define the horizontal axis of the global frame.

#### 3) Orientation Estimation

With the sensor-to-segment alignment and the common global frame for every IMU, it is possible to reconstruct the movement of the chest, arm and hand by starting in the static neutral pose. Orientation estimation is usually done by integrating the angular velocity over time to get the angular change of the sensor. However, this method causes integration drift due to noise and offsets in the gyroscope measurements. Therefore, sensor fusion algorithms were used to correct both the inclination and heading of the sensor. In this study, a Madgwick filter [35] was used to compensate the IMU inclination errors due to integration drift, by fusing data from the gyroscopes and accelerometers. Zero-angular velocity updates are used to reduce errors in the orientation estimation. This error correction technique was based on the work of Kirking *et al.* [36]: if the norm of the angular velocity is below 3°/*s*, the sensor is considered to be static. By using a linear interpolation between two periods where the sensor is static, the effects of the drift and offset in the gyroscope measure were eliminated during the movement, reducing the integration drift when estimating the sensor’s orientation.

The orientation estimation of each sensor ${S}$ is given in quaternion form and converted to a rotation matrix [37]. Finally, the body segment’s orientation relative to the global frame is calculated by aligning the sensor’s frame to the anatomical frame using the sensor-to-segment calibration parameters.

\begin{equation*} ^{G}\textrm {R}_{Seg}(t) = \hspace {0.1cm} ^{G}\textrm {R}_{S}(t) \hspace {0.1cm} ^{S}\textrm {R}_{Seg}\tag{3}\end{equation*}

where $^{G}\textrm {R}_{Seg}(t)$ is the orientation of the limb segment at time $t$ relative to the global frame.

#### 4) Joint Angle Extraction

The arm joint angles can be represented as the angle between the anatomical axis aligned with the respective limb segments. The anatomical frames seen from the global frame are represented by the collumns of the rotation matrices calculated in the orientation estimation.

For the elbow, wrist and fingers flexion/extension angles, the angle was calculated between the axes of the two adjacent limb segments. The longitudinal axis of the more distal segment of the joint was first projected to the flexion/extension plane, with the normal vector corresponding to the axis in the medio-lateral direction, of the more proximal segment of the joint. This excludes measuring ulnar or radial deviations or lateral movements of the fingers that do not correspond to flexion or extension of the segment. The shoulder flexion/extension and abduction/adduction was calculated by relating the upper arm frame to the sternum frame. For the flexion angle, the axis aligned with the upper arm is projected onto the body’s sagittal plane, which corresponds to the $xz$-plane of the sternum’s frame. The abduction angle is calculated by projecting the same upper arm axis onto the frontal plane, which is defined as the $zy$-plane of the sternum’s frame. The projected vector $\vec {v^{p}}$ is given by:

\begin{equation*} \vec {v^{p}} = \vec {v} - \frac {\vec {v} \cdot \vec {n}}{||\vec {n}^{2}||}\vec {n}\tag{4}\end{equation*}

where $\vec {v}$ is the axis of the segment’s frame and $\vec {n}$ is the plane normal vector. The angle $\theta $ between two vectors $\vec {v_{1}}$ and $\vec (v_{2})$ is given by:

\begin{equation*} \theta = atan2\left({\frac {||\vec {v_{1}} \times \vec {v_{2}}||}{\vec {v_{1}} \cdot \vec {v_{2}}}}\right)\tag{5}\end{equation*}

where $atan2$ is the four-quadrant inverse tangent, outputting angles between $-\pi $ and $\pi $. Details on the individual joint angles calculation can be found in appendix V.

### C. Experimental Design

#### 1) Participants

Ten moderately affected (FMA-UE between 34 and 54) chronic stroke survivors, recruited from the University Hospital Zurich, Switzerland were included in this study. All participants suffered from a unilateral lesion resulting from either an ischemic or a hemorrhagic stroke. The participants included in this study were required to be at least 18 years old and to have a diagnoses of stroke in the chronic stage (>6 months). All participants had to have stroke-associated impairments of the upper limb (FMA-UE lower than 60) and be at least partially able to move the arm against gravity and to perform finger movements for basic grasp function. Subjects were excluded if they had pre-existing impairments of the upper limb e.g. orthopaedic impairments, severely increased muscle tone and sensory deficits. Participants were also excluded if severe communication or cognitive deficits cause inability to follow the procedures and give informed consent, or if there were contraindications on ethical ground. The FMA-UE test was performed before the start of the protocol to characterize the motor impairment level. Detailed demographic characteristics are presented in Table 2.

**TABLE 2:** Demographic and Clinical Characteristics of the Participants (N = 10)

Characteristic	Value
Sex (male/female)	6/4
Age median (IQR) in years	61.5 (57.5 - 70.0)
BMI median (IQR)	26.5 (23.7 - 29.2)
Arm length median (IQR) in cm	68.5 (62.6 - 76.6)
Dominant hand (left/right)	0/10
Time since stroke median (IQR) in months	31.5 (20.3 - 63.8)
Affected side (left/right)	5/5
FMA-UE median (IQR) / max	33.5 (32.3 - 34.8) / 66
Arm subscore / max	22.0 (22.0 - 24.8) / 36
Wrist subscore / max	6.0 (5.5 - 6.8) / 10
Hand subscore / max	9.5 (6.8 - 11.0) / 14
Coordination subscore / max	2.5 (2.0 - 3.8) / 6

All participants gave written informed consent in accordance with the declaration of Helsinki. The cantonal ethics in Zurich approved the experimental protocol prior to start of the study (Req-2019-00417).

#### 2) Experimental Protocol

Before placing the distributed sensing system, the participants were assessed using the full FMA-UE test on the affected and non-affected side by a physiotherapist. The experimental protocol was performed on both limbs separately, starting with the non-affected limb. After donning of the system, the sensor-to-segment calibration procedure was performed. The therapist helped the participant maintaining the static postures and performing the dynamic movements. Each posture was measured for at least five seconds. The protocol with the system in place consisted of performing four items from the FMA-UE test to examine movements within and out of the pathological synergies. Each item consist of one or more tasks that are scored on a 0 to 2 scale ($0=$ can not perform, $1=$ performs partially, $2=$ performs fully) by a physiotherapist [38]:

1) *FMA-UE* item A2: movement within the synergies, where the subject is asked to raise the hand from the contralateral knee to the ipsilateral ear from extensor synergy (shoulder adduction/ internal rotation, elbow extension, forearm pronation) to flexor synergy (shoulder abduction/ external rotation, elbow flexion, forearm supination). The movement is scored in a 0 to 2 scale based on the performance of the shoulder retraction, elevation, abduction of ang 90 and external rotation and on elbow flexion and forearm supination (max 12 points).
2) *FMA-UE* item A3: movement mixing the flexor and extensor synergies, where the subject is instructed to flex the shoulder ang 90 and maintain the elbow fully extended (ang 0). The maximum score of 2 points is achieved when there is no shoulder abduction or elbow flexion, immediate abduction or elbow flexion results in 0 points, abduction or elbow flexion during movement results in 1 point,
3) *FMA-UE* item A4: movement out of the pathological synergies, where the subject is asked to abduct the shoulder ang 90, keep the elbow fully extended (ang 0) and the forearm pronated. The maximum score of 2 points is achieved this the subject performs this movement flawless. Immediate supination or elbow flexion results in a score of 0 points, during movement in 1 point.
4) *FMA-UE* item B: intended to evaluate the range of wrist flexion and extension. The subject is asked to flex the shoulder (at least ang 70) and perform wrist flexion and extension movements while keeping the elbow and fingers extended (ang 0). A smooth full active range of motion results in maximum score of 2 points, no volitionally movement in 0 points and a limited range of motion in 1 point.

The subjects were seated and performed three repetitions of each item. This procedure is repeated for the affected arm.

### D. Data Analysis

#### 1) Feature Extraction

**FIGURE 2.**
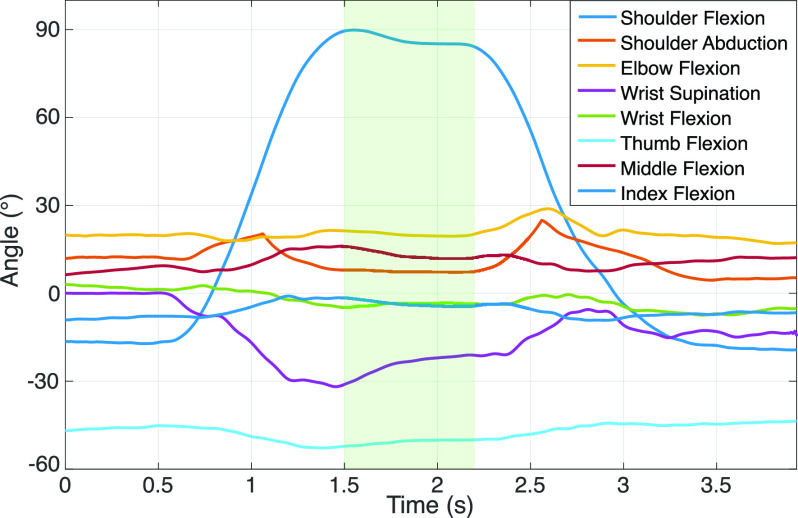
Arm joint angles measured in one participant during task 2 of the experimental protocol (item A3). The green shaded area indicates the time interval where the arm is in the target position.

To characterize the subjects’ performance, different features were extracted from measurements. An example of the joint angle estimation for one of the tasks can be seen in Figure 2. The figure represents the upper limbs joint angles of a non-affected arm. A positive angle indicates flexion or abduction of a joint, and a negative angle represent extension or adduction. The static neutral pose (with the elbow and fingers extended and pointing down) is considered to be the zero for the joint angles. The features used to evaluate the subjects performance are the joint angles measured in the target position of each task (green shaded area in Figure 2).

#### 2) Statistical Analysis

The participants were divided into two different main groups with different levels of impairment (group 1 and group 2). The groups were created based on the FMA-UE arm subscore. The more affected group (group 1) included subjects with a score of 22 or less ($N = 6$, FMA-UE arm subcore median and interquartile range (IQR): 22 (22 - 22)). The less affected group (group 2) was composed by the remaining participants ($N = 4$, FMA-UE arm subscore median and IQR: 25.5 (24.5 - 28.5)). Within each group, the data from the affected (AF) and non-affected (NAF) arms was separated, creating in total four different groups for the statistical analysis (NAF-1 - non-affected arm of group 1; AF-1 - affected arm of group 1; NAF-2 - non-affected arm of group 2; and AF-2 - affected arm of group 2).

Two different statistical tests were applied. First, the Wilcoxon signed rank test was used to analyse differences between the extracted features within the same impairment level group (differences between NAF-1 and AF-1 and between NAF-2 and AF-2). Second, the Mann-Whitney U test was used to check differences between the measurements of groups 1 and 2, of the same arm (differences between NAF-1 and NAF-2 and between AF-1 and AF-2).

Both tests are non-parametric due to the small sample size and the low evidence of normally distributed data. All statistic analysis was done with a significance level of 0.05.

### E. Validity Tests

Before the experiments with stroke subjects, the validity of the distributed system and the experimental protocol was tested using a rigid arm model and a healthy subject test. The rigid arm model, consists of a wooden frame composed of a body and two wood parallelepiped pieces, around the size of a human arm. The “upper arm” wooden piece is connected to the body via two eye hooks. The “lower arm” piece is connected to the upper arm via a hinge. It simulates the shoulder joint, allowing for flexion and abduction, and an elbow joint that is able to flex from a fully extended posture (elbow flexion angle of ang 0). The rigid model test simulated multiple ang 90 movements for the shoulder flexion, shoulder abduction, elbow flexion, wrist flexion, extension, pronation and supination. The wrist movements were simulated by attaching the sensor to an additional wooden block and manually moving it in the desired direction. The results of the rigid model test correspond to the angles calculated using the orientation estimation and feature extraction methods. The estimated reconstructed angles of the shoulder flexion (ang 87), abduction (ang 90), elbow flexion (ang 90) and the wrist flexion (ang 88) and extension (ang 87) show errors less than ang 3 in the target position of the tested ang 90 movements. The wrist supination/pronation (ang 102/ ang 99) tests with the rigid model show an overestimation of the joint angle of around ang 10. During the tests, the overall angles of the other joints are significantly small (mean and standard deviation ${-1}^{\circ}\pm 2$). The finger flexion angles show a higher deviation than the other joint angles during the tests (${4}^{\circ}\pm 3$).

The second test involved a healthy subject (Male, Age: 23, BMI: 22.1, Arm Length: 73 $\mathrm { \text {c} \text {m} }$), since healthy individuals have the ability of performing more controlled movements, for the global frame definition, the sensor-to-segment protocol and the actual movements of the protocol. These tests were approved by the University of Twente faculty ethics committee. The measured joint angles in the target position showed some variations in the different repetitions. However, the mean joint angles were close to the instructed angles. In movements 2 and 3 (shoulder abduction and shoulder flexion), the mean of the measured angles of these joints are ${89}^{\circ}\pm {1}^{\circ}$ and ${92}^{\circ}\pm {4}^{\circ}$, close to the desired 90° angle. The subjects were instructed to fully extend the elbow in tasks 2, 3 and 4. The measured mean elbow flexion angles are close to 0° (${-2}^{\circ}\pm {5}^{\circ}$). The negative values can be caused by overextension of the elbow. Other joint angles are consistent throughout the protocol, where the highest standard deviation of all the measured features was 15°, corresponding to the middle finger flexion angle in movement 4.

## III. Results

All ten stroke subjects were able to complete the experimental protocol. Data from the thumb IMU was discarded for one subject due to malfunctions of the sensor. In one repetition of the fourth item of the protocol, the IMU data had to be excluded due to recording errors. In total, 119 movements were analysed (30 repetitions of tasks 1, 2 and 3 of the protocol and 29 of task 4).

**FIGURE 3.**
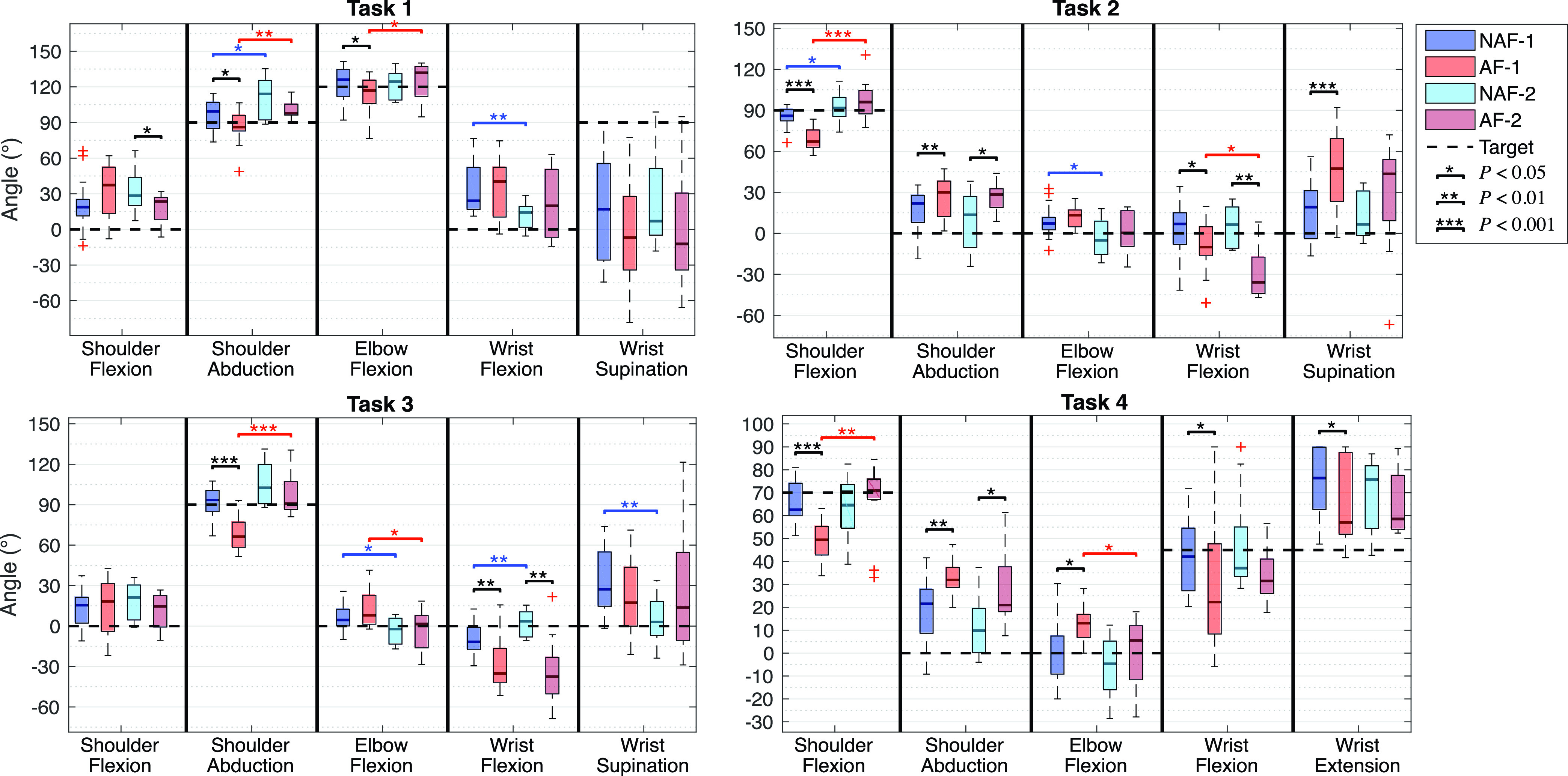
Boxplots (median and IQR) of the target angles for the most significant joints evaluated in the four tasks of the protocol in the four groups. The black dashed lines represent the desired targets for the task. Significant differences between arms of the same group are indicated with a black line. Significant differences between the same arm of different groups are indicated with a blue (NAF) or red (AF) lines. $\ast P < 0.05$; $\ast \ast P < 0.01$; $\ast \ast \ast P < 0.001$.

Figure 3 shows the joint angles in the target position measured in the four different groups when subjects performed the experimental tasks. The median and the IQR ($25^{th}$ and $75^{th}$ percentiles) of the measured relevant joint angles of the group are shown in the form of boxplots. The presented joint angles allow the visualization of possible pathological synergistic behaviour in the proximal limb segments (shoulder and elbow). The considered relevant joints were based on the scoring criteria of the FMA-UE [17]. The desired target joint angles for each task are marked with dashed lines in the figure. It can be seen that there are significant differences ($P < 0.05$) between the four data groups, mainly at the shoulder and elbow level. Depending on the task, results show that the desired target position is harder to reach when the more affected subjects perform the task with the affected arm (AF-1). The shoulder flexion angle of AF-1 is significantly lower in task 2 and task 4 and the shoulder abduction is significantly lower in task 3 compared to the non-affected arm (NAF-1) and less affected group (AF-2). The elbow is significantly more flexed in the more affected groups when compared to the respective less affected groups (AF-1 vs AF-2 and NAF-1 vs NAF-2).

**FIGURE 4.**
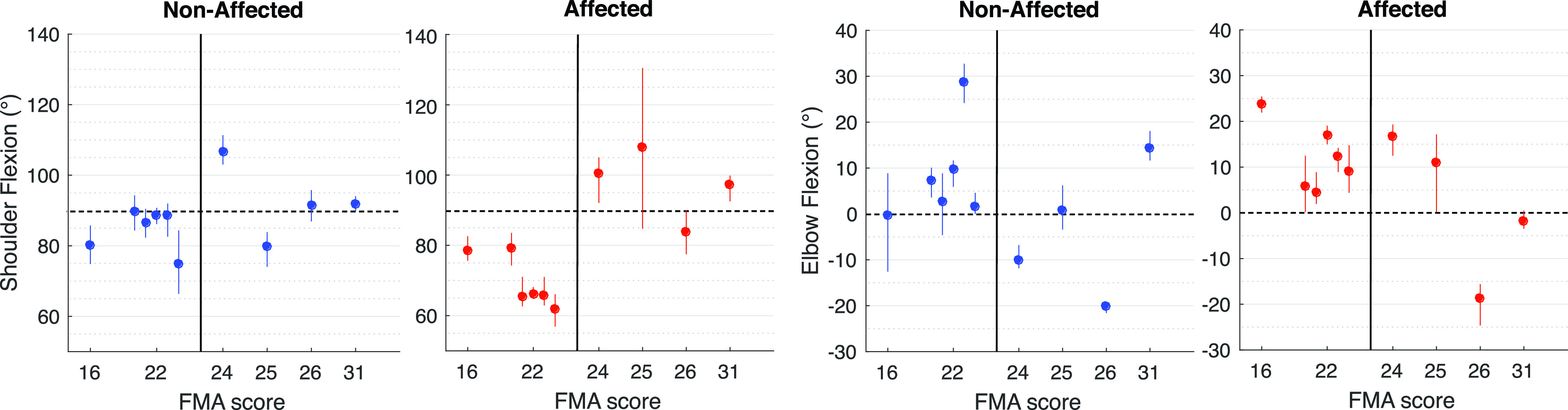
Relation between the shoulder and elbow flexion of the AF (red) and NAF (blue) arms and the FMA-UE arm subscore during task 2 (FMA-UE item A3) of the protocol. The black dashed lines represent the desired targets for the task.

The relation between the FMA-UE score and the measured features can be seen in Figure 4. The shoulder and elbow flexion angles of the AF and NAF arms of all subjects on the target posture of task 2 (shoulder flexion movement) are plotted against the FMA-UE score of the participants. The figure presents the mean of the joint angles measured on the three repetitions of each subject. Similarly to what is observed in Figure 3, more affected subjects have a smaller shoulder flexion angle and a higher elbow flexion. The most affected subject (with FMA-UE of 16) appears to have a higher shoulder flexion angle than the subjects with an FMA-UE score of 22. In addition, the five participants with a score of 22 have different arm postures.

## IV. Discussion

Measuring kinematics during clinical motor function assessment scales has not been performed extensively in the past. The majority of studies use kinematic measures in activities of daily living or while performing functional tasks, such as reach-to-grasp tasks [13], [20], [39], [40]. Some studies used inertial measurement units while performing simple movements that include flexion/extension, abduction and supination of limb segments [30], [40]. In this work, a new distributed sensing system was developed and tested in an upper limb model, healthy and stroke subjects. The kinematic analysis with the developed distributed IMU measurement system is a minimally obstructive technology that uses IMUs for quantification of arm function. By performing the arm-related tasks of the clinical scale, the presence of the described pathological synergies can be analysed via features that describe the movement patterns of the patients. The results presented in Figure 3 and the results from the statistical analysis show significant differences between the postures of the affected and non-affected limbs. However, some might not be clinically relevant.

The measured joint angles during the first task show a similar behaviour in the shoulder and elbow flexion of the AF and NAF arms. The shoulder abduction angle is significantly lower in the more affected group of subjects when compared to the less affected group (a difference of about ang 20), both in the NAF and AF arms. However, the median is higher than the desired target angle. These findings suggest that when the movement is performed within the flexor synergy (abduction of the shoulder accompanied by elbow flexion and wrist supination and flexion), the kinematic differences might not be related to the presence of the flexor synergy, but to muscle weakness or paresis, causing the inability to abduct the shoulder against gravity. The lower shoulder and elbow range of motion is only noticed between the affected and non-affected arms of the more impaired group, indicating that less affected subjects have indeed fewer effects of paresis. The wrist supination angles are below the desired target angle in all groups, having a very high variance in the results. Effects of pathological coupling are inconclusive in the wrist joint.

The second task is intended to mix the flexion and extension synergies. The results show that the shoulder flexion angle is significantly lower in the affected side of the more impaired group of subjects. The measured joint angles indicate that there is a higher elbow flexion angle in the AF side of both groups of subjects when compared to the NAF of the respective group. This behaviour becomes clinically relevant due to the high median difference between groups, the AF arm of the most affected group shows a shoulder flexion and an elbow flexion median angle ang 20 and ang 10 lower than the NAF arm respectively. The subjects were clearly instructed to perform the movement with their elbow completely extended. The cause of a lower shoulder flexion can be related to weakness or a compensation strategy to avoid elbow flexion. It is possible that they prioritize the elbow angle and not the shoulder flexion. The shoulder abduction angle is higher in the affected arm of both groups of subjects, which is a manifestation of the flexor synergy, where the shoulder flexion is accompanied by involuntary abduction. Finally, the wrist supination angle only shows to be significantly higher in the affected arm of the less impaired group when compared to the non-affected arm of the same group, which is also a behaviour present in the described pathological flexor synergy [8].

The third task (shoulder abduction) shows a significant difference between the affected and non-affected limbs. When the task is done with the affected arm of the more affected group, the shoulder is not sufficiently abducted in order to reach the desired target angle, only being able to reach around ang 20 below. The pathological synergy behaviour is, however, not clearly visible in this task since the elbow flexion angle does not show significant differences between arms of the same group, and the median value difference between arms of the different groups of subjects is smaller than ang 10. Similarly to the previous task, it is possible that subjects prioritize the extension of the elbow and abduct less the shoulder.

The fourth task, intended to evaluate wrist function, shows expected results, the wrist flexion and extension is significantly higher in the non-affected arm when compared to the affected limb of the more impaired group. The shoulder flexion angle in this movement is not well-defined for the subjects. They are instructed to flex the shoulder ang 70, which is not easy for the subject to determine where it is. Even so, it appears that the shoulder flexion angle is significantly lower in the AF arm of the more affected group (approximately ang 20 smaller than the target). The elbow flexion is significantly higher in the affected limb, as well as the shoulder abduction, which follows the observations of pathological synergies. Both these joint angles appear to have a median ang 15 higher in the AF-1 group when compared to the NAF-1 and NAF-2. It has been observed in the past that wrist and finger motor function is mostly the last to recover [10], [41].

The statistical tests done between the upper extremities of the more and less affected groups (group 1 and 2 respectively) suggest that the variation of the level of impairment is related to the features presented in this study, and also to the presence of a more severe pathological synergistic behaviour. A significant decrease in the shoulder flexion or abduction and elbow flexion of group 1 to group 2 can be noticed in tasks 2 and 3, showing signs of joint coupling. The more impaired group of subjects shows a significantly lower shoulder flexion in the affected and non-affected arms of the more impaired group when compared to the correspondent side of the less impaired group in task 2 (shoulder flexion movement). However, the difference between the affected arm’s elbow flexion angle of group 1 and 2 is not significant, where the median difference is smaller than ang 10. In task 3 (shoulder abduction movement), the shoulder abduction angle is significantly smaller and the elbow flexion is higher in the more affected group. The elbow flexion difference is seen between both the affected arms and the non-affected arms of the two groups, whereas the shoulder abduction difference is only seen between the affected arms of groups 1 and 2. This could suggest that the ipsilesional upper extremity is also affected by stroke. This finding is supported by previous research [42], where it was found that the ipsilesional arm also suffers from less severe motor impairments after stroke. Another explanation would be that the subject does not perform the movement well with the non-affected arm due to factors not related to stroke, and only due to age-related weakness or low flexibility in the joints. Nevertheless, the results show that the difference between the shoulder abduction of the AF and NAF arms in the same group of subjects is significant in group 1 but not in group 2, indicating that the less impaired subjects perform the task similarly with both arms, having less severe pathological muscle coupling. The decreased coupling is also seen in the fourth task in the elbow flexion and wrist flexion/extension angles. Less impaired subjects have a lower elbow flexion angle at the affected side when the wrist is flexed or extended. The wrist range of motion, however, does not vary significantly between the arms of the same group. However, care needs to be taken when comparing group 1 and group 2 since the deviation of the participants into the more affected group 1 with a FMA-UE score of 22 or less, is made for illustrative purposes and not an established method.

Several objective kinematic features have been shown to have a strong correlation with clinical scales [13], [30], [40], [43], [44]. The relation between the measures shown in the present study and the level of impairment, although it follows the theoretical aspects that qualify motor function in stroke patients, does not have a high statistical power due to the low sample size and the small range of impairment severity in the population. Figure 4, shows that the results strongly varies among the subjects and only have a limited relation with FMA-UE arm score. E.g. the most affected subject (FMA-UE arm sub score of 16) appears to be able to flex the shoulder ang 15 more than less affected subjects (with a score of 22). Despite this, the coupling effect in the affected arm is noticeable and relates better with the clinical score, where the most affected subject shows the highest elbow flexion angle and the less affected show smaller angles. Furthermore, the inter-individual differences shown in Figure 4 are large and the variability does not clearly relate to the FMA-UE score. This might be caused by the more fine grain scale of the IMU system compared to the FMA-UE, in which a score of 1 covers a wide range between 0 (can not perform) and 2 (performs fully). This may mean that the sensing system provides complementary info to the more general FMA-UE clinical score. However, clinicians still need to assess whether this complementary info is of clinically relevant value.

In this study, the only features used in the movement analysis consisted of joint angles. Several other types of features related to the movement profile can indicate the presence of motor function deficits, like movement smoothness and velocity profiles [30], [40], [43], [45]. These features were not explored, however, the data acquired allows for additional analysis. The measurement of the joint angles at different time points of the task can be of interest as well. In the tasks performed in this experiment, the FMA-UE score is based on the range of motion of the joints and also at the moment where the coupling of the shoulder and elbow starts. This moment is hard to be visually identified in less impaired stroke patients, as well as the smoothness and the velocity profiles. The sensors used in the distributed measurement system developed for this study have the ability to measure these features. Another approach is to study the presence of pathological synergies as a response to shoulder abduction loading on the range of monition and the hand and finger dexterity as described by Ellis *et al.* [27]. This has been studied, using the same system, by handling objects with certain weights and thus functionally applying shoulder abduction loading [46].

Although, the subjects reported no experience of movement limitations caused by the system, donning and calibrating takes a considerable amount of time. Further studies could lead to a set of clinical relevant features, that could be measured using a reduced set of optimal placed IMUs decreasing the obtrusiveness of the distributed measurement system by reducing the donning and calibration time.

Although the results of this study followed expected behaviours of stroke patients and the described coupling patterns of the pathological synergies, some limitations were identified. Different sources of errors could have a negative impact in the arm orientation estimation. The quality of the measurements was assumed to be acceptable based on trials performed on a rigid model of the arm and on healthy subjects. From the validity tests with the model, it can be concluded that the system’s configuration and processing methods can measure shoulder and elbow joint angles with a maximum deviation from the desired target of ang 3. When the experimental protocol is performed by the healthy subject the deviations from these joints are higher (maximum of ang 5). The differences between the real and measured joint angles can be explained by several factors. Firstly, the movement performed is never perfectly the same in different repetitions. Secondly, the sensor-to-segment calibration protocol and the global frame definition may not be perfectly executed, causing deviations between the measured joint angles and the actual arm posture. The therapist helped the stroke subjects maintaining the required postures or performing these dynamic movements, however, it was not always possible to perform these movements correctly. Furthermore, the sensor-to-segment calibration relies on an equal body shape for each subject. The effect of this assumption is visible Figure 4, where the subject with a score of 26 appears to have a very high negative elbow flexion of ang −20, in both the AF and the NAF arms. To some extent, this could be due to over extension, as known for the subject with a FMA score of 24 which showed a ang −10 elbow flexion. However, the ang −20 on both the AF and the NAF seems to be caused by errors in the assumptions made during calibration, i.e., when the elbow is not completely extended in the calibration postures a full extension looks like overextension. Task 2 and 4 in Figure 3 also indicate that some subjects have a high negative elbow angle on both AF and NAF side. The cause might be that some subjects have more body volume, which affects the anatomical axis measurements during the static postures of the sensor-to-segment calibration protocol. In this particular case, in item 7 of the protocol, the inclination of the upper arm caused by a non-fully adducted posture creates misalignments between the gravity vector measured by the sensor and the anatomical x-axis of the limb segment. More calibration movements would increase the accuracy in the joint angle estimation and the estimated angles would correspond better to reality. Another source of error is the lack of consistency of the initial position for each task. The kinematic reconstruction is based on the orientation of the initial posture, defined by the orientation of the sensors related to the global frame before the task execution. This orientation is used to reset the orientation of the sensors and reduce drifts caused by gyroscope and accelerometer bias, noise and possible external factors that affect the measurements (like temperature and sudden movements). If the subject deviates a lot from the initial posture defined in the beginning of the experiment, the orientation estimation of the task will be prone to errors. To accurately assess the validity of the proposed methods, a reference system should be used, such as optical motion trackers. Nevertheless, the tests done using the rigid arm model and on the healthy subject show that the system and the analysis methods are reliable. Furthermore, the system is capable of visualising the expected synergies. However, the sources of error have to be taken into account and a further investigation should be done to validate the proposed methods. The usage of the distributed IMU system developed in this work can increase the impairment’s evaluation quality and eliminate the subjective perspective of the human eye. The complementary use of the system during the FMA-UE test is of interest since it objectively measures the observations done by the therapist during the assessment and provides a detailed analysis of the movement.

## V. Conclusion

The presented pilot study showed that the developed distributed measurement system is capable of distinguishing movements of the affected and non-affected upper extremities of stroke subjects using inertial sensors. The distributed IMU system can objectively measure kinematic features, such as joint angles, to assess motor function of subjects when they perform tasks of the FMA-UE test. The pilot study on 10 chronic stroke subject showed that the system can identify the presence of pathological muscle couplings and measure features related to the pathological synergies present in the movement of stroke subjects. Also, the severity of the pathological coupling reflected by the arm joint angles is related to the level of impairment of the subjects, where the more affected subjects show the presence of more severe pathological synergistic behaviour and weakness in the shoulder, elbow and wrist joints. The system has the potential to provide more detailed information with respect to the FMA-UE sub-score, since the individual joint angles show no clear relation with the FMA-UE score. Despite the fact that the sensor-to-segment calibration in stroke subject needs to be evaluated in more detail, it is concluded that the distributed measuring system shows high potential as a useful tool for assessing motor function of the upper extremity of stroke subjects.

## Appendix A Joint Angles Calculation

### Shoulder Joint Angles

The shoulder flexion/extension angle is calculated by firstly projecting the x-axis of the upper arm frame $\vec {x_{ua}}$ onto the $zx$-plane of the sternum, by taking equation 4, with $\vec {v} = \vec {x_{ua}}$ and $\vec {n} = \vec {y_{st}}$. The flexion/extension angle $\theta $ is calculated by taking the angle between $\vec {x_{ua}}^{p}$ and $-\vec {z_{st}}$. If $\theta = {0}^{\circ}$ means that the shoulder is straight next to the body, pointing down. A positive $\theta $ corresponds to shoulder flexion and a negative $\theta $ to shoulder extension. The shoulder abduction/adduction angle is calculated by projecting $\vec {x_{ua}}$ to the $zy$-plane of the sternum’s frame ($\vec {n} = \vec {x_{st}}$). A positive angle indicates abduction, a negative angle corresponds to adduction.

### Elbow Joint Angles

The elbow flexion/extension angle is taken by projecting $\vec {x_{la}}$ onto the $zx$-plane of the upper arm’s frame. A positive angle means elbow flexion. If $\theta = {0}^{\circ}$, it indicates that the elbow is fully extended. Negative values for this joint angle mean that there is elbow over-extension.

### Wrist Joint Angles

The wrist flexion/extension angle is calculated the same way as the elbow, but by projecting the x-axis of the hand’s frame $\vec {x_{h}}$ onto the $zx$-plane of the lower arm’s frame. A positive angle indicates wrist flexion and a negative angle wrist extension. If the angle is ang 0, it means that the wrist is in the neutral position. Wrist pronation/supination is calculated differently, since there is no plane that accompanies the movement of the hand in order to correctly measure the desired angle. The lower arm pronates and supinates along with the wrist, so comparing its frame to the wrist’s frame will not represent the true supination/pronation angle. For this case, the joint angle corresponds to the integration over time of the $x$ component of the gyroscope of the hand IMU. The gyroscope data is first aligned to the segment by multiplying it by the correspondent sensor-to-segment calibration rotation matrix. A positive angle indicates that the wrist is pronated and a negative angle represents a wrist supination. When $\theta = {0}^{\circ}$, the wrist is in neutral position.

### Finger Joint Angles

The index and middle fingers flexion/extension angles are calculated by projecting the finger’s x-axis, $\vec {x_{i}}$ and $\vec {x_{m}}$, onto the $zx$-plane of the hand’s frame. Then, the angle between the x-axis of the hand $\vec {x_{h}}$ and the projected axis of the fingers $\vec {x_{i}}^{p}$ and $\vec {x_{m}}^{p}$ is calculated. The thumb flexion/extension angle is calculated by projecting $\vec {x_{t}}$ onto the $xy$-plane of the hand, and calculating the angle between $\vec {x_{h}}$ and $\vec {x_{t}}^{p}$. A positive angle indicates finger flexion and a negative angle represents extension.
